# A Treatise on Food and Diet, with Observations on the Dietetical Regimen Suited for Disordered States of the Digestive Organs, &c.

**Published:** 1843-10-01

**Authors:** 


A Treatise on Food and Diet, with Observations on the Dietetical Regimen suited for Disordered States of the Digestive Organs; and an Account of the Dietaries of some of the principal Metropolitan and other Establishments for Paupers, &c. &c. &c. By *Jonathan Pereira*, M.D. F.R.S. & L.S. London. Longman, &c. &c. 1843.

We already possessed in our language several works on the Materia Alimentaria, viz. Cullen's celebrated works on that subject, Dr. Paris's deservedly popular work "on Diet," with many others of various degrees of merit. The present treatise, however, differs from its predecessors in many particulars. In the first place it contains a tolerably full account of the *Chemical Elements* of the food; 2dly, it is remarkable for the increased space devoted to the consideration of *Alimentary Principles*, after which the subject of *Compound Aliments* is considered. This plan of separately considering *Alimentary Principles* and *Compound Aliments* the author has adopted from Tiedemann. The work is divided into Two Parts. In the first part we have the chemical elements of the food, the *Alimentary Principles of*

*the Food* and *Compound Aliments* discussed at considerable length. In the *second Part*, the subject of DIET, more properly so called, is treated of, viz. the Digestibility of Food—the Times of Eating—Dietaries for various Classes of Persons, as Dietaries for Children—Dietary for the Naval Service—for the Army—for Paupers—for the Sick—for the Insane, &c. We shall without further preamble proceed to Part I.

### PART I.—OF FOODS.

The substances employed as food by man consist of certain compound bodies termed *Alimentary Principles*, which, by their mixture or union, constitute our ordinary foods: these may be called *Compound Aliments*.

Thus meat (a compound aliment) consists principally of fibrine, albumen, gelatine, hæmatosin, fat and water (alimentary principles). Alimentary principles are themselves compound substances. They consist of two, three, four, or more *simple or undecompounded bodies*, called *elements*. These are the *Chemical Elements*. Thus fibrine (an alimentary principle) is composed of carbon, hydrogen, nitrogen, oxygen, phosphorus and sulphur (chemical elements).

With respect to the author's plan of arrangement, he proposes to consider—

1st. The Chemical Elements of Foods.
2. The Alimentary Principles.
3. The Compound Aliments.

A living body, says the author, has no power of forming elements, or of converting one elementary substance into another; and it therefore follows that the elements of which the body of an animal is composed must be the elements of its food. The essential constituents of the human body are thirteen; and the same, therefore, must be the elements of our food.

*Chemical Elements of the Food of Man.*

| | | | |
|---|---|---|---|
| 1. Carbon. | 5. Phosphorus. | 8. Chlorine. | 11. Potassium. |
| 2. Hydrogen. | 6. Sulphur. | 9. Sodium. | 12. Magnesium. |
| 3. Oxygen. | 7. Iron. | 10. Calcium. | 13. Fluorine. |
| 4. Nitrogen. | | | |

These substances he now proceeds to notice separately.

1. *Carbon.*—In the pure state carbon constitutes the *diamond*. In its impure forms carbon constitutes *plumbago* (black-lead), and *charcoal* (animal and vegetable).—Carbon is an essential constituent of every living or organised tissue, both vegetable and animal; hence it is a necessary ingredient of food, and Nature has accordingly supplied it in the aliment which she has provided for all living beings in the early stage of their existence. Thus it is an element of the organic substances composing seeds, and from which the embryo plant derives its first nutriment. The yolk of egg (the food of the embryo chick), and milk, (the food of the young mammals during the first period of existence after birth) also contain it. Different kinds of foods have been found to contain different quantities of this element.

The quantity of carbon consumed, in the form of food, by different individuals and at different times, varies very much. Among the circumstances, to which such variations may be attributed, we may reckon age, sex, peculiarities (individual or national), temperature and density of the air, occupation, and amount of clothing. According to Liebig, an adult taking moderate exercise consumes about $15\frac{1}{3}$ oz. avoirdupois of carbon daily—all this quantity of carbon, or nearly all of it, is thrown out of the system by the lungs and skin in the form of carbonic acid, the carbon uniting with the oxygen, which is derived from the air, either by the lungs or skin, or both. By this union of carbon with oxygen, in whatever part of the system it is effected, heat must be evolved. According to Despretz, one pound of pure charcoal evolves, by its combustion in oxygen gas, sufficient heat to raise the temperature of 78 lbs. of water from 32° Fahr. to 212° Fahr.

Liebig has shewn that, by the conversion of starch or sugar into fat, oxygen is supplied to the system; and that, by the union of this disengaged oxygen with carbon (from the bile, for instance), heat is developed. Suppose one equivalent of carbonic acid $CO_2$, and 7 equivalents of oxygen, $O_7$, to be abstracted from one equivalent of starch, $C_{12}H_{10}O_{10}$, we have, in the residue, the empirical formula for fat, $C_{11}H_{10}O$.

*Relative Composition of Starch and Fat.*

| | | | |
|---|---|---|---|
| 1 eq. Starch . . | $C_{12}H_{10}O_{10}$ | 1 eq. Fat . . . . | $C_{11}H_{10}O$ |
| | | 1 eq. Carb. acid . | $C\ O_2$ |
| | | 7 eq. Oxygen . . | $O_7$ |
| | | | $C_{12}H_{10}O_{10}$ |

The oxygen, thus presumed to be separated from the starch, can only be disengaged in the form of either carbonic acid or water, or of both; and, consequently, must have combined with carbon or hydrogen, or both. Liebig has adduced several reasons for presuming that heat must attend the formation of carbonic acid under these circumstances. "Thus," says he, "in the formation of fat, the vital force possesses a means of counteracting a deficiency in the supply of oxygen, and, consequently, in that of the heat indispensable for the vital process."

Under ordinary circumstances, the carbon necessary for the support of animal heat is supplied by the food, but when food is withheld, the fat of the body is consumed; its carbon is converted into carbonic acid, and its hydrogen into water. Experience has shewn that the heat of the blood is the same in all climates and in all atmospheric temperatures. Hence a larger quantity of combustible matter is required in cold climates and cold weather, than in hot climates and in warm weather—and hence, also, the necessity of a more liberal supply of food in cold weather. "He who is well fed," observes Sir John Ross,* "resists cold better than the man who is stinted, while the starvation from cold follows but too soon a starvation in food. This, doubtless, explains in a great measure the resisting

* Narrative of a Second Voyage in Search of a North-West Passage; p. 200. London, 1835.

powers of the natives of these frozen climates, their consumption of food, it is familiar, being enormous, and often incredible."

Those persons who are at all familiar with the accounts which have been published regarding the natives of the arctic regions, must be well aware of their gormandising powers. Captain Sir W. E. Parry, (Second Voyage for the Discovery of a North-west Passage,) states, that as a matter of curiosity, he one day tried how much food an Esquimaux lad would consume, if freely supplied. "The under-mentioned articles were weighed before being given to him; he was twenty hours in getting through them, and certainly did not consider the quantity extraordinary."

| | lbs. | oz. |
|---|---|---|
| Sea-horse flesh, hard frozen .. | 4 | 4 |
| Ditto, .. boiled .. .. | 4 | 4 |
| Bread and bread-dust .. .. | 1 | 12 |
| Total .. | 10 | 4 |

| The fluids in fair proportion, viz. | | |
|---|---|---|
| Rich gravy soup, | 1¼ | pint. |
| Raw spirits .. | 3 | wine glasses. |
| Strong grog .. | 1 | tumbler. |
| Water .. .. | 1 | gallon 1 pint. |

Sir John Ross, in his *Narrative*, states, that an Esquimaux "perhaps eats twenty pounds of flesh and oil" daily.

The kinds of food which theory points out as best suited for the natives of these colder climates, scil. foods abounding in carbon and hydrogen, as fats and oils, which contain from 66 to 80 per cent. of carbon, are precisely those used by them.

Our author considers it erroneous in Liebig to ascribe the voracity of the inhabitants of the colder regions to the influence of cold only.

The Hottentots and Bushmen of Southern Africa, are well known to be beastly gluttons; a circumstance which cannot be ascribed to the temperature of their climate, while "the inhabitants of the Alpine regions of Southern Europe, demand no such extravagance of food, nor are even the people of Lapland and the Northern extremity of Norway conspicuous for such eating; as is not less true of the Icelanders."* The author conceives, that this voracity is ascribable, in part at least, to some instinct or propensity exercised by some portion of the brain. It is obvious, from what has been now stated, that much less heat is evolved when there is a deficiency of food; it is equally evident that, in tropical climates, and even in more temperate climates, during the Summer, a smaller quantity of food suffices to keep up the temperature of the body; and, under such circumstances, substances containing a less proportion of carbon, are better adapted for preserving the health.

Liebig ascribes the frequency of diseases of the liver in hot seasons and tropical climates, to the accumulations of carbon in the system. If, the external temperature being high, we continue to consume large quantities of food, there will be an excess of carbonaceous matter in the system. The effect of high temperature, excess of food and want of exercise, on the state of the liver, is well shewn in the goose. The same reasoning will apply, at least in part, to the frequency of hepatic disease among Europeans residing in tropical climates, and who continue to employ the

* Sir John Ross, *op. supra cit.*

same dietetical system which they followed in their own more temperate climate.

*Hydrogen.*—Hydrogen, like carbon, being an essential constituent of every organised tissue, is, therefore, a necessary ingredient of the food of every living being, both vegetable and animal. In reference to the quantity of hydrogen they contain, alimentary principles may be arranged in three groups: the first containing those substances whose oxygen and hydrogen are in the same relative proportion as in water; the second, including those whose oxygen is to the hydrogen in a less proportion than in water, or which contain an excess of hydrogen; and the third, comprehending those whose oxygen is to the hydrogen in a proportion greater than is necessary to form water, or which possess an excess of oxygen.

*Relative Quantity of Hydrogen and Oxygen in Alimentary Principles.*

| Group 1. Principles whose Oxygen and Hydrogen are in the same ratio as in Water. | Group 2. Principles containing an excess of Hydrogen. | Group 3. Principles containing an excess of Oxygen. |
|---|---|---|
| Acetic Acid.<br>Starch.<br>Sugar.<br>Gum. | Oil.<br>Alcohol.<br>Malic Acid.<br>Fibrine, Albumen, Caseine } Animal and Vegetable.<br>Gluten.<br>Gelatine. | Pectine.<br>Citric Acid.<br>Tartaric Acid. |

GROUP 1. *Alimentary Principles whose Oxygen and Hydrogen are in the same ratio as in Water.*—The substances of this group may be regarded as *hydrates of carbon,* since they consist of carbon and water, (or its elements). Their composition is as follows:—

*Hydrates of Carbon.*

| | |
|---|---|
| Acetic acid . . . . . . | 12 C + 9 water. |
| Starch . . . . . . . . | 12 C + 10 water. |
| Cane sugar . . . . . | 12 C + 10 water + 1 water. |
| Gum . . . . . . . . . | 12 C + 10 water + 1 water |
| Sugar of milk . . . . | 12 C + 10 water + 2 water. |
| Grape sugar . . . . . | 12 C + 10 water + 4 water. |

It is obvious that these foods can yield carbon only to be oxidated in the system, since the hydrogen is already in combination with oxygen. This, therefore, is a sufficient explanation of the fact mentioned by Liebig, that the graminivorous animals expire a volume of carbonic acid equal to that of the oxygen inspired; in other words, there is no loss of oxygen, since one volume of carbonic acid gas contains a volume of oxygen. In a state of nature, a large proportion of the food of these animals consists of principles (starch, sugar, and gum,) whose hydrogen is saturated with oxygen. In no other way can we account for the fact just referred to; for, as Liebig correctly observes, "at the temperature of the body, the affinity of hydrogen for oxygen far surpasses that of carbon for the same element," and, therefore, the return of an equal volume of carbonic acid by expiration is an evidence that there was a want of hydrogen for the oxygen to combine with.

GROUP 2. *Alimentary Principles, whose Oxygen is to the Hydrogen in a less proportion than in Water, or which contain an excess of Hydrogen.*—This group includes both nitrogenised and non-nitrogenised food. If we suppose the oxygen of those principles to be combined with hydrogen in the ratio to form water, there will remain, for each, an excess of hydrogen; the amount of which, however, varies in different substances. The author here gives a table, constructed on this view, shewing the excess of hydrogen which each principle contains, the amount of carbon in each being calculated to be the same:—

*Alimentary Principles containing excess of Hydrogen.*

| | | | |
|---|---|---|---|
| Malic acid (anhydrous) | = 48 C + 18 | water | + 6 H. |
| Fat (lard) . . . . | = 48 C + 4.5 | water | + 38.5 H. |
| Alcohol . . . . . | = 48 C + 24 | " | + 48 H. |
| Proteine . . . . . | = 48 C + 14 | " | + 22 H + 6 N. |
| Albumen . . . . | = 48 C + 14 | " | + 22 H + 6 N + S + P. |
| Fibrine . . . . . | = 48 C + 14 | " | + 22 H + 6 N + 2 S + P. |
| Caseine . . . . . | = 48 C + 14 | " | + 22 H + 6 N + S. |
| Gelatinous tissues, tendons . . . | = 48 C + 18 | " | + 23 H + 7.5 N. |
| Chondrine . . . . | = 48 C + 20 | " | + 20 H + 6 N. |

The ultimate changes which these constituents of food undergo in the system, are the conversion of the carbon into carbonic acid, and the hydrogen into water. "It signifies nothing," says Liebig, "what intermediate forms food may assume, what changes it may undergo in the body, the last change is uniformly the conversion of its carbon into carbonic acid; and of its hydrogen into water. The unassimilated hydrogen of the food, along with the unburned or unoxidised carbon, is expelled in the urine, or in the solid excrements."

GROUP 3. *Alimentary Principles, whose Oxygen is to the Hydrogen in a proportion greater than is necessary to form Water.*—None of the substances of this group, which includes pectine (vegetable jelly) and some vegetable acids, are nitrogenised. The following table represents the composition of these principles, on the supposition that the hydrogen is combined with oxygen in the ratio to form water, the calculation being made for the same amount of carbon in each:—

*Alimentary Principles containing an excess of Oxygen.*

| | | | |
|---|---|---|---|
| Pectine . . . . . | 12 C + 8.5 | water | + 5 O. |
| Citric acid (dry) . . . | 12 C + 5 | " | + 6 O. |
| Tartaric acid (dry) . . | 12 C + 6 | " | + 9 O. |

All the hydrogen and part of the carbon of these principles are, therefore, in combination with oxygen.

3. *Oxygen.*—Oxygen is a necessary ingredient of our food. The following table, taken from Liebig, gives the relative proportions of carbon and oxygen in several alimentary principles:

| | | | | |
|---|---|---|---|---|
| In Fats (on an average) .. | 120 | equiv. of Carbon.. . | 10 | equiv. of Oxygen. |
| " Fibrine, albumen & caseine | 120 | " | 36 | " |
| " Starch .. .. .. | 120 | " | 100 | " |
| " Cane sugar .. .. | 120 | " | 110 | " |
| " Gum .. .. .. | 120 | " | 110 | " |
| " Sugar of milk .. .. | 120 | " | 120 | " |
| " Grape sugar .. .. | 120 | " | 140 | " |

As the carbon and hydrogen of the food are ultimately for the most part thrown out of the system in combination with oxygen—that is, in the form of carbonic acid and water—it follows that those foods which contain a small proportion of oxygen only must consume a greater amount of atmospheric oxygen than those which possess a larger quantity of this element. Hence it is clear that the quality of the food must affect the activity of the respiration. Dr. Fyfe has found the consumption of oxygen to be greatly reduced by the employment of vegetable diet.

The influence exercised by matters taken into the stomach on the process of respiration is well illustrated in the case of the vegetable salts of potash or soda. If the acetate, citrate, or tartrate of either of these alkalies be swallowed, the salts suffer partial decomposition in its passage through the system—its base can be detected in the urine; but its acid has disappeared, and is replaced by carbonic acid. To effect this, a considerable quantity of oxygen must be consumed. In the case of acetate of potash, no less than eight equivalents of oxygen are required to convert the carbon of every atom of acetic acid into carbonic acid.

*Conversion of Acetic Acid into Carbonic Acid and Water.*

| | | | |
|---|---|---|---|
| 1 equiv. acetic acid | $C_4O_3\ H_3$ | 4 equiv. carbonic acid | $C_4O_8$ |
| 8 equiv. oxygen.. | — $O_8$ — | 3 equiv. water .. .. | —$O_3\ H_3$ |
| Total .. | $C_4O_{11}H_3$ | Total .. | $C_4O_{11}H_3$ |

When we take an ordinary effervescing draught composed of tartaric acid and bicarbonate of soda, there is developed, by their mutual reaction, tartrate of soda, which, in its passage through the system, suffers decomposition. Its tartaric acid disappears, and is converted into carbonic acid and water by means of oxygen.

*Conversion of Tartaric Acid into Carbonic Acid and Water.*

| | | | |
|---|---|---|---|
| 1 equiv. tart. acid, | $C_4O_5\ H_2$ | 4 equiv. carbonic acid, | $C^4O_8$ — |
| 5 equiv. oxygen .. | —$O_5$ — | 2 equiv. water .. .. | —$O_2\ H_2$ |
| Total .. | $C_4O_{10}H_2$ | Total .. | $C_4O_{10}H_2$ |

Now the eight equivalents of oxygen in the first case, and the five equivalents in the latter instance, must be derived either from the organism or from the atmosphere. But, as Liebig justly observes, there is no evidence presented by the organism in itself that any of its constituents have yielded so large a quantity of oxygen; and we have a right, therefore, to infer, that it must have been derived from the air; and that these salts, in their passage through the lungs, appropriate to themselves the necessary amount of oxygen. But do they appropriate that which, if they were not present, would be otherwise employed in the organism? or do they consume an extra quantity of oxygen? We have no precise data on which we can satisfactorily answer this question. Liebig asserts, that they must consume a part of the oxygen, which would otherwise unite with the constituents of the blood; and "the immediate consequence," he observes, "of this, must be the formation of arterial blood in less quantity; or, in other words, the process of respiration must be retarded."* The necessity of this conclusion of Liebig our author cannot see; he has already shewn that the amount of oxygen consumed by respiration, is modified by the quality of the food, and considers it not improbable, therefore, that the passage of the above-mentioned salts through the lungs may occasion a temporary augmented consumption of oxygen; but the evidence for or against this notion is yet to be adduced.

*Nitrogen* or *Azote*.—Nitrogen is distinguished from the three preceding substances by the indifference which it manifests to enter into chemical combination with other bodies. It is an essential constituent of every animal tissue. Fat and water are non-nitrogenised components of the animal body, but they are not organised or living substances. It is obvious, therefore, that for the development, growth, nutrition, and renovation of living animal parts, nitrogen is essential; and accordingly we find, that Nature has supplied it in the food which she has furnished for the young animal; it being a constituent of the albumen of the yolk of the egg, (the food of the embryo chick), and of the caseine of the milk, (the aliment of the young mammal). Several vegetable and animal substances contain no nitrogen, and various articles of food contain it in different proportions.

*Nitrogenised foods* alone being considered as capable of conversion into blood and of forming organised tissues, these have been called by *Liebig* the *plastic elements of nutrition*. What then is the use of the substances in the food which are destitute of nitrogen, and which we know to be essential to animal life? Liebig has shown that these are exhausted in the production of animal heat, being converted into carbonic acid and water: in other words, their use is to support the process of respiration by yielding their carbon and hydrogen, the oxidation of which is attended with the development of heat. These non-nitrogenised substances he therefore calls *elements of respiration*.

---

* May not the refrigerant effects of these salts be explained in this manner, and their consequent utility in febrile diseases?—REV.

| *Nitrogenised Substances,* or *Plastic Elements of Nutrition.* | |
|---|---|
| Vegetable | Fibrine. |
| ——— | Albumen. |
| ——— | Caseine. |
| Animal | Flesh. |
| ——— | Blood. |

| *Non-nitrogenised Substances,* or *Elements of Respiration.* | |
|---|---|
| Fat, | Pectine, |
| Starch, | Bassorine, |
| Gum, | Wine, |
| Cane Sugar, | Beer, |
| Grape Sugar, | Spirits. |
| Sugar of Milk, | |

The opinion that nitrogenised foods alone nourish the tissues, is supported by several arguments. The first is, that *as the animal tissues contain nitrogen as one of their essential constituents, and as this element cannot be created in the system, it must be derived from either the food or the atmosphere; but, as it is not absorbed from the atmosphere in the vital process, it must be obtained from the food.*

The entire force of this argument depends on the truth of the assertion, that "no nitrogen is absorbed from the atmosphere." Various experiments have been instituted by various physiologists on this point, and with the most discordant results. Müller says, that "the conclusion to be deduced from all these experiments seems to be, that, during respiration, nitrogen is both absorbed and exhaled by the blood." In fact, some of the best physiologists of modern times admit the absorption of nitrogen. Hence, then, till it be demonstrated that "no nitrogen is absorbed from the atmosphere," the above argument goes for nothing; it is in fact a downright begging the question.

The second argument is, *that non-nitrogenised foods alone are incapable of supporting animal life.*—"This argument has not, however, much weight, since it is well known, that an exclusive diet of nitrogenised alimentary principles (gluten excepted) is also incapable of supporting animal life. Fibrine, albumen, or gelatine, taken separately, does not support life; even the artificial mixture of these principles is insufficient to preserve life—for dogs, thus fed, ultimately die with all the signs of complete inanition."

On reflection, we are very much inclined to think that the author has entirely mistaken the meaning of the question here; we think the import of it is, that no foods, but such as contain nitrogen, are capable of nourishing the tissues, or that foods, entirely destitute of nitrogen, are incapable of nourishing the tissues. Now Magendie, Tiedemann, and Gmelin, have satisfactorily proved the truth of this; the first found, that dogs fed exclusively on sugar and water died in from thirty-one to thirty-four days; the two latter experimenters found, that geese fed on sugar and water, or gum and water, or starch and water, died in from sixteen to twenty-four days.

Instead of the author's saying that "the second argument has not much weight," he should have said, that it is no argument at all; in as much as it is identical with, and just equivalent to, the question at issue—both the question, and the argument adduced in its support, are, what logicians call, identical propositions; they also come under the title of exclusive propositions; now, it is well known, that every exclusive proposition may be converted into an equivalent exceptive, and *vice versa;* now, by thus converting these two exclusive propositions (the question and the argument) into their equivalent exceptives, we shall obtain two exceptive propositions precisely the same even to the very letter.

*Question.*—Nitrogenised foods alone nourish the tissues.

*Equivalent exceptive.*—No foods, *except nitrogenised foods,* nourish the tissues.

*Argument.*—Non-nitrogenised foods alone are incapable of nourishing the tissues.

*Equivalent exceptive.*—No foods, except nitrogenised foods, nourish the tissues.

Here the two exceptive propositions, obtained by converting the two exclusives, are identical to the very letter—and therefore the question and the argument adduced to support it, are equivalent, and so the author has allowed himself to be caught in that vulgar sophism, called a "begging of the question." In fact, he should have seen that the question regarded a matter of fact, and not a matter of reasoning; Magendie, Tiedemann, and Gmelin saw the same thing, and therefore very properly had recourse to *experiment* and not to *logic,* for the ascertaining of its truth.

Not less strange is the reason given by the author for considering the "argument," as he calls it, as possessing but little weight; "since it is well known, he says, that an exclusive diet of nitrogenised alimentary principles is also incapable of supporting animal life." The reason assigned for the weakness of the "argument" has about as much to do with this unfortunate "argument" itself, as the present place of the moon in her orbit has to do with "the Repeal of the Union." What we find fault with in the author is, his acknowledging that to be an argument (though a weak one, he admits) which is identical with the *questio probanda,* and next, in assigning as a proof of such weakness a reason which is no reason at all. The alogy which makes so conspicuous a figure in the writings of some of our modern medical authors, affords abundant proof of the necessity of requiring of those who aspire to the rank of M.D.'s, some substantial certificates that they have received somewhat of a liberal education.

The third argument is, that *the food of all animals, herbivorous and carnivorous, contains nitrogenised matters, identical in composition with the principal constituents of the blood and organised tissues of the animal body; and, therefore, the carbon, sugar, and starch, and the carbon and hydrogen of the fats and oils, are not required for the production of blood.*

The connexion between the consequence in this proposition and the antecedent is anything but striking. By the way, we should much like to know, whether these "arguments are indebted for their existence as such, to the author; if so, among the many other charges which may be brought against him concerning them, the charge of infanticide, or at least that of child-exposure, is not the least.

The fourth argument is, that *the quantity of nitrogenised food, which herbivorous animals consume, is amply sufficient for the growth and development of their organs, and for the supply of waste.*

The opinions recently advanced with respect to the uses of nitrogenised and non-nitrogenised foods in the animal economy, are thus briefly stated by the author:—

1. Nitrogenised foods are alone capable of conversion into blood, and of forming organised tissues.

2. Nitrogenised foods which contain proteine, as albumen, fibrine, caseine and gluten, alone form the albuminous and fibrinous tissues.

3. Gelatine is incapable of conversion into blood; but it may perhaps serve for the nutrition of the gelatinous tissues (cellular tissue, membrane and cartilage).

4. Non-nitrogenised foods support the process of respiration by yielding carbon, and, in some cases, hydrogen, to be burnt in the lungs, and thereby to keep up the animal temperature.

5. Some of the non-nitrogenised foods contribute to the formation of fat, the carbon and hydrogen of which are ultimately burnt in the lungs, and thereby develop heat.

6. With the exception of the substance of cellular tissue, of membranes, and of the brain and nerves, all the organic materials of which the animal body is composed are derived from vegetables, which alone possess the property of producing compounds of proteine.

The author now states a few circumstances which appear to him to raise some difficulties to the unqualified admission of the opinions above referred to.

1. When benzoic acid, a non-nitrogenised substance, is taken into the stomach, it appears in the urine in the form of hippuric acid. This hippuric acid is probably formed by the elements of the benzoic acid, with the addition of those of lactate of urea.

| | | | |
|---|---|---|---|
| 1 equiv. Urea . . | $C^2N^2H^4O^2$ | 2 equiv. Crystallised Hippuric acid . . | $C^{36}N^2H^{18}O^{12}$ |
| 1 equiv. Lactic acid | $C^6—H^4O^4$ | | |
| 1 equiv. Benzoic acid | $C^{28}—H^{10}O^6$ | | |
| Total . . | $C^{36}N^2H^{18}O^{12}$ | | |

It cannot, therefore, be doubted, "that a non-azotised substance, taken in the food, can take a share, by means of its elements, in the act of transformation of the animal tissues, and in the formation of a secretion." Consequently, the possibility of the conversion of non-nitrogenised foods into nitrogenised constituents of the animal body, does not appear by any means improbable.

2. Liebig's explanation of the uses of nitrogenised and non-nitrogenised foods does not account for the fact stated by the Commissioners of the French Academy, that while fibrine, albumen, and gelatine, taken together or separately, are incapable of supporting animal life, gluten from wheat or maize is alone sufficient to satisfy complete and prolonged nutrition. As fibrine, albumen, and gluten, are said to be identical in composition, their nutritive powers ought to be equal.

The other objections to the recently advanced opinions, already alluded to, we shall here pass over. That non-nitrogenised substances are intended by nature to constitute part of the food of man and other animals, there are abundant proofs, as we find them in the aliments supplied by nature for animals during the first period of existence. Thus, in the yolk of egg we have a fixed oil—in milk we have sugar and butter, both non-nitrogenous principles. The craving of animals, too, for these substances, and the fact already mentioned, that nitrogenised food alone cannot support life, afford ample proof that those principles are essential to health and life. .. .. ..

Alcohol is classed among the elements of respiration; and it cannot be doubted that it undergoes some changes in the animal economy. When taken into the stomach it is absorbed, and gets into the circulating mass. Now, how does it get out of the system? certainly not by the bowels, urine, or skin. A portion of it escapes by the lungs, and is recognizable by its odour in the breath; but the quantity in this way thrown out of the system is comparatively small, and is certainly disproportionate to that often swallowed. Moreover, it is principally when the quantity taken is very large, that it is most recognisable in the breath;—when, in fact, the formation of respiration is very imperfectly formed. What, then, becomes of it? By itself it cannot form tissues, since it is deficient in some of their essential ingredients, namely, nitrogen, sulphur, and phosphorus; and there is no reason to suppose that it contributes even in part, to the renovation of tissues. Liebig's suggestion, that it is burnt in the lungs, and thereby converted into carbonic acid and water, appears very plausible. To convert it into these substances, oxygen only is required: as may be thus shewn:—

| | | | |
|---|---|---|---|
| Alcohol . . . . . | $C^4H^6O^2$ | Carbonic acid . . . . | $C^4O^8$ |
| Oxygen . . . . . | ——$O^{12}$ | Water . . . . . . | $H^6O^6$ |
| Total . . | $C^4H^6O^{14}$ | Total . . | $C^4H^6O^{14}$ |

By its oxidation in the lungs it must evolve caloric, and thus, when used in moderation, it serves to support the temperature of the body. This use of it in the animal system, appears to have been entirely overlooked by the Tee-totallers. Though alcohol thus supports the temperature of the body, it must be admitted to be an obnoxious fuel. Its volatility and the facility with which it permeates membranes and tissues, enable it to be rapidly absorbed; and, when it gets into the blood, it exerts a most injurious operation, before it is burnt in the lungs, on the brain and liver.

*Nutritive Equivalents.*—Several authors have attempted to form a scale of nutritive equivalents. Boussingault has suggested one, founded on the quantity of nitrogen contained in foods.

*Scale of Nutritive Equivalents.*

| *Substances.* | *Equivs.* | *Substances.* | *Equivs.* |
|---|---|---|---|
| Wheat flour . . . . . . | 100 | Rice . . . . . . . . . | 177 |
| Wheat . . . . . . . . . | 107 | Buckwheat , . . . . . . | 108 |
| Barley meal . . . . . . . | 119 | Maize . . . . . . . . . . | 138 |
| Barley . . . . . . . . . | 130 | Horse-beans . . . . . | 44 |
| Oats . . . . . . . . . . | 117 | Peas . . . . . . . . . | 67 |
| Rye . . . . . . . . . . | 111 | | |

It will be seen that, in this table, 44 parts of horse-beans, or 67 of peas, are represented as being equal in nutritive power to 100 parts of wheat flour. This, as our author justly remarks, cannot be correct. Liebig admits, that though lentils, beans and peas, surpass all other vegetable food in the quantity of nitrogen they contain, yet that they possess but small value as articles of nourishment, because they are deficient in the component parts of the bones (subphosphate of lime and magnesia); they satisfy the appetite without increasing the strength.

*Phosphorus.*—This is a constituent of both animals and vegetables—it is an essential ingredient of albumen and fibrine and of all tissues composed of these principles. Nervous matter also contains it, as does also the brain—the bones too contain it—it is detected in the spermatic fluid and in the ovary. It being thus a necessary ingredient of the animal body, it must, of course, be an element of the food of animals. It is a constituent of the blood, the flesh and the bones of animals employed by men as food. Phosphorus is a constituent of most vegetable substances, being found in the ashes of plants, principally in the form of an earthy phosphate (lime or magnesia).

*Sulphur.*—This is a constituent of both animals and vegetables. Fibrine and albumen, and all tissues composed of these substances, contain it. The existence of sulphur in the very many animal substances in which it is found, serves to explain the evolution of sulphuretted hydrogen and hydrosulphuret of ammonia, by putrefying animal substances; excrement, for example. That sulphuretted hydrogen is evolved in privies is proved by its darkening the white paint, and by its blackening silver articles (watches, spoons, &c.) which have accidentally fallen into the night-soil. Game, when very high, will sometimes discolour the silver fork used in eating it.

Sulphur is thrown out of the system in various excretions. Thus, the urine contains sulphates, in part formed by the action of the oxygen of the arterial blood on the sulphur of the metamorphosed tissues. In the saliva we meet an alkaline sulpho-cyanide; and, in consequence of the presence of this salt, the saliva possesses the property of reddening the sesqui-salts of iron. Metallic matter kept in the mouth becomes discoloured by the action of sulphur on it. The gold plates used to support artificial teeth, become incrusted with a film of metallic sulphuret. The system derives its sulphur from animal and vegetable substances used as food. Thus flesh, eggs and milk contain it. Vegetable fibrine (as of corn), vegetable albumen (as of almonds, nuts, cauliflowers, turnips, &c.), and vegetable caseine (as of peas and beans), contain it—it is chiefly abundant in the *cruciferæ*.

An infusion of *white* mustard strikes a blood-red colour with the persalts of iron, owing to the presence of sulpho-sinapisine. By this character white mustard is readily distinguished from black mustard. Both kinds of mustard flour charred in a tube evolve a sulphuretted vapour, which blackens paper moistened with a solution of acetate of lead .In the same way sulphur may be detected in cabbage, potatoes, and many other vegetable foods. It is from such substances that the sulphur of our system is derived.

*Iron.*—This is a constituent of most, if not all, organised beings. The quantity they contain is, however, very small. Iron is an essential constituent of the blood, though, according to some recent experiments by Scherer, it is neither essential to hæmatosin nor necessary to the colour of the blood. The beneficial effects of chalybeates in the disease called anæmia, in which the blood is found to contain a smaller quantity of iron than in a state of health, favours the notion that the healthy colour of the fluid is in some way connected with the amount of iron contained in it. According to Denis, 1000 parts of the blood-corpuscles yield 2 parts of per or sesqui-oxide of iron. But, as the relative proportions of serum and blood-corpuscles are subject to considerable variation, it follows that the quantity of iron contained in a given weight of blood cannot be constant. The quantity of sesqui-oxide of iron obtained from 1000 parts of blood, varies, from 0·128 to 0·346, according to the authority just quoted. Liebig assumes the existence of a much larger quantity of iron in the blood. Iron is a constituent of the hair. Black hair contains most of this metal; white hair the least.

Most articles of food contain iron. It is a constituent of the blood found in meat. Veal must contain less of it than beef, since calves are usually bled copiously previous to death, by which an anemic state is induced. In the yellow fat of the yolk of egg this metal may be detected. Milk likewise contains iron in a state of phosphate. Traces of iron have been detected in most vegetable foods.

*Chlorine.*—This elementary substance is a constituent of the blood, the gastric juice, and several of the excretions, as the urine, saliva, tears and fæces. In the blood and the excretions it exists in combination with sodium, while in the gastric juice it is found combined with hydrogen, and thereby constituting hydrochloric acid.

As the chlorine of the blood is constantly being consumed in the formation of the gastric juice and secretions, it requires to be frequently renewed. Hence it is an indispensable constituent of our food; and is taken into the system in the form of the chloride of sodium or common salt, which contains 60 per cent. of chlorine.

*Sodium.*—This is a constituent of the blood, the animal tissues and the secretions. Owing to its presence, the ashes of animal substances (feathers, bristles, hairs, &c.) possess the property of communicating a yellow tinge to flame. This metal is taken into the system, chiefly in the form of a chloride, which contains 40 per cent. of the metal. This salt is used at our table as a condiment, and is a constituent of most animal foods. It is not an ordinary constituent of plants, unless they grow in the neighbourhood of the sea or other salt water—minute quantities of it are found in most of our common waters. Sodium is expelled from the system both in the form of chloride and of oxysalt. In the urine of carnivorous animals it exists in the form of sulphate and phosphate of soda.

*Calcium.*—This metal is a component part of all animals. In the higher classes it exists principally in the form of subphosphate of lime. Thus, the bones of the vertebrata contain this salt mixed with a small portion of carbonate of lime. But the shells and crusts of invertebrated animals, as lobsters, oysters, &c. consist of carbonate of lime principally, but mixed with a little subphosphate of lime. Muscles, nervous matter, liver, &c. and indeed all the animal solids, as well as the blood, contain calcium in the form of subphosphate of lime. The calcium of our system is derived from the animal, vegetable and mineral substances which we consume as food. Thus bones, flesh, viscera, blood, and milk of animals, yield us this metal —most vegetables also contain it—another source of calcium is common water, which usually contains both bicarbonate and sulphate of lime.

*Magnesium.*—Small quantities of this metal are found in the blood, teeth, bones, nervous matter, thyroid gland, and other parts of the body. It exists in combination with oxygen and phosphoric acid, and often with ammonia also. It is a constituent of both vegetable and animal foods.

*Potassium.*—Minute traces of potassium exist in blood, the solids, and several of the secretions of animals—this substance is a constituent of both animal and vegetable food—most plants which grow inland contain it; thus it is found in grapes and potatoes.

We now come to consider the contents of *Chap* 2, which treats of Alimentary Principles.

The first chapter we have just seen treats of simple or undecompounded bodies. Two or more of these simple bodies form, by their union with each other, certain compound substances termed *Alimentary Principles*, or *Simple Aliments;* and by the combination of the latter our ordinary foods, called compound aliments, are formed.

Some alimentary principles contain but two elements, as water. Others contain three, as sugar and fat. Proteine is formed of four elements, while fibrine and albumen contain six. Some aliments, as water and common salt, are derived from the mineral kingdom: others are obtained from the organized kingdom. Dr. Prout, in his work *On the Nature and Treatment of Stomach and Urinary Diseases*, arranges alimentary principles into four classes, scil. the *aqueous*, the *saccharine*, the *oleaginous*, and the *albuminous*.

The author is induced by chemical and physiological considerations to adopt a different classification, and proposes the following:—

*Classes of Alimentary Principles.*

| | |
|---|---|
| 1. The Aqueous. | 7. The Acidulous. |
| 2. The Mucilaginous or Gummy. | 8. The Alcoholic. |
| 3. The Saccharine. | 9. The Oily or Fatty. |
| 4. The Amylaceous. | 10. The Proteinaceous. |
| 5. The Ligneous. | 11. The Gelatinous. |
| 6. The Pectinaceous. | 12. The Saline. |

1. *The Aqueous Alimentary Principle.*—With one or two very trifling exceptions water, in some form or other, seems essential to vitality. A very large proportion of the human body is water. The blood contains about 80 per cent., the flesh about 74 per cent. of water. So that we may lay it down that the entire human body contains about three-fourths of its weight of water. But as by evaporation, as also by the processes of secretion and exhalation, part of this fluid is wasted or consumed, the necessity of the use of water as a drink becomes obvious. It is, in fact, more necessary to our existence than solid food. The sources of the water contained in the system are the aqueous drinks which we consume, as also the moisture contained in most of the solid substances employed as food. Water serves several important purposes in the animal economy: firstly, it repairs the loss of the aqueous part of the blood, caused by evaporation and the action of the secreting and exhaling organs; secondly, it is a solvent of various alimentary substances, and therefore, it assists the stomach in the act of digestion, though, if taken in very large quantities, it may have an opposite effect, by diluting the gastric juice; thirdly, it is probably a nutritive agent. It is not actually demonstrated that water yields up its elements to assist in the formation of organised tissues; that it may do so, however, is not improbable. From Liebig's observations it would appear that the hydrogen of vegetable tissues is derived from water; and it is not probable that the higher orders of the organised kingdom should be deficient in a power possessed by the lower orders.

The water which constitutes an essential part of the blood and of the living tissues, assists in several ways in carrying on the vital processes. It is from water that the tissues derive their properties of extensibility and flexibility. Lastly, this fluid contributes to most of the transformations which occur within the body. As a solvent it serves to aid digestion, and also to effect other changes. It is probable that the conversion of uric acid into urea, by the action of oxygen, is effected by the agency of water, which holds the acid in solution; for in animals which drink much water, no uric acid, but urea only, is found in the urine; while in birds which seldom drink, and in snakes, uric acid predominates.

*Conversion of Uric Acid into Urea.*

| | | | | | | | | | |
|---|---|---|---|---|---|---|---|---|---|
| 1 eq. Uric acid | $C^{10}$ | $N^4$ | $H^4$ | $O^6$ | 2 eq. Urea . . | $C^4$ | $N^4$ | $H^8$ | $O^4$ |
| 4 eq. Water . . | — | — | $H^4$ | $O^4$ | 6 eq. Carbonic acid | $C^6$ | — | — | $O^{12}$ |
| 6 eq. Oxygen . | — | — | — | $O^6$ | | | | | |
| Total. . . | $C^{10}$ | $N^4$ | $H^8$ | $O^{16}$ | Total . . . | $C^{10}$ | $N^4$ | $H^8$ | $O^{16}$ |

Water, considered as a dietetical remedy, may be regarded under a two-fold point of view;—first, with respect to its *quantity;* secondly, in reference to its *quality.*

In febrile and inflammatory diseases an almost unlimited use of aqueous fluids is admitted under the various names of *slops, diluents,* &c. &c. They quench thirst, lessen the stimulating quality, and augment the fluidity of the blood—in some maladies, however, it is necessary to restrict the quantity of the fluids taken; it becomes necessary, in fact, to adopt *a dry diet,* as when we desire to keep down the quantity of the circulating fluid (as in valvular disease of the heart), or to prevent thinness of the blood (as in aneurysm of any of the great vessels, where our only hope of cure depends on the coagulation and deposition of fibrine within the aneurysmal sac), or when we wish to repress excessive secretion (as of urine, in diabetes).

With respect to *quality*, the waters furnished us by nature may be divided into three classes: viz. 1st. Common waters, or those employed as drinks, or for dressing food, or other purposes of domestic economy. 2nd. Sea water. 3rd. Mineral waters. Distilled water is usually obtained from the first of these.

1. *Common waters.*—Under this head are included the waters commonly known as *rain, spring, river, well* or *pump, lake* and *marsh waters.*

*a. Rain water.*—Of this we need say nothing.

*b. Spring water.*—This is rain water, which, having percolated through the earth, re-appears at the surface of some declivity. During its passage it almost always takes up some soluble matters, which of course vary according to the nature of the soil.

*c. River water.*—This is a mixture of rain and spring water. When deprived of the matters which it frequently holds in suspension, its purity is usually considerable. The carbonate of lime, one of the ordinary solid constituents of river water, is held in solution by carbonic acid, forming bicarbonate of lime. By boiling, this acid is expelled, and the carbonate of lime is precipitated on the sides of the vessel, constituting the *fur* of the tea-kettle, and the *crust* of boilers.

Decomposing organic matter, in suspension or solution, is found in every river water in a greater or less proportion. Ordinarily the quantity is insufficient to act injuriously; but it cannot be doubted that water strongly impregnated with it must be deleterious. In those cases in which its operation has been unequivocally recognised, it has manifested itself by the production of dysentery. Its influence in a milder form is attended with slight relaxation of the bowels. This decomposing organic matter consists principally of the exuviæ of animal and vegetable substances.

*d. Well water.*—This is water obtained by sinking wells. As it is commonly raised by sinking pumps, it is frequently called *pump water.* The constituents of ordinary well-water are similar to those of river-water above mentioned; but the earthy salts (especially the bicarbonate and sulphate of lime) are found in much greater quantity. It usually decomposes and curdles soap, for which reason it is called *hard-water*, to distinguish it from river and other waters, which are readily miscible with soap, and which are termed *soft water.* The hardness of water depends on certain earthy salts, the most common of which is sulphate of lime. By the mutual action of this salt and soap, double decomposition is effected: the sulphuric acid unites with the alkali of the soap, setting free the fatty acids, which unite with the lime to form an insoluble earthy soap. Hard water is a less perfect solvent of organic matter than soft water; hence in the preparation of infusions and decoctions, and for many domestic purposes, as tea-making and brewing, it is inferior to soft water; and, for the same reason, it is improper as a drink in dyspeptic affections. It proves injurious also in urinary deposits.

Though the purest waters are the most wholesome, yet very pure water is possessed of one very dangerous quality: viz. that of rapidly corroding lead, and thereby acquiring an impregnation of this metal. Distilled water has no action on lead, provided the air be excluded, but when this is admitted, a thin white crust of carbonate and hydrate of the oxide of lead is speedily formed. Now, it is very remarkable that the neutral salts, usually found in spring water, impair the corrosive action of water and air, and thus exercise a protecting influence. To the presence of saline matter, therefore, is to be ascribed the comparative infrequency of the plumbeous impregnation of water kept in leaden cisterns or transmitted through leaden pipes. All salts do not possess an equally protective influence, the carbonates and sulphates being most, the chlorides (muriates) the least, energetic of those saline substances commonly met with in spring waters. Rain and other pure kinds of water which contain but minute portions of these protecting salts, readily acquire an impregnation of lead from roofs, gutters, cisterns, or pipes, made of this metal. Galvanic action is another cause of lead being acted on by water. Water impregnated with lead, in the way above alluded to, possesses the following properties:—By exposure to the air it becomes covered with a thin white film, and the vessel in which it is contained becomes lined with a thin white incrustation of a pearly lustre. This crust, dissolved in acetic acid, yields a solution which is rendered blackish brown by sulphuretted hydrogen, and yellow by either iodide of potassium, or bichromate of potash.

The following conclusions, drawn by Dr. Christison, as to the employment of lead-pipes for conducting water, are of considerable importance, and therefore deserve especial attention.

"1. Lead-pipes ought not to be used for the purpose, at least where the distance is considerable, without a careful examination of the water to be transmitted.

"2. The risk of a dangerous impregnation with lead is greatest in the instance of the purest waters.

"3. Water, which tarnishes polished lead when left at rest upon it in a glass vessel for a few hours, cannot be safely transmitted through lead-pipes without certain precautions.

"4. Water which contains less than about an 8000th of salts in solution, cannot be safely conducted in lead-pipes without certain precautions.

"5. Even this proportion will prove insufficient to prevent corrosion, unless a considerable part of the saline matters consist of carbonates and sulphates, especially the former.

"6. So large a proportion as a 4000th, probably even a considerably larger proportion, will be insufficient, if the salts in solution be in a great measure muriates.

"7. It is I conceive right to add, that in all cases, even though the composition of the water seems to bring it within the conditions of safety now stated, an attentive examination should be made of the water after it has been running for a few days through the pipes. For it is not improbable that other circumstances, besides those hitherto ascertained, may regulate the preventive influence of the neutral salts.

"8. When the water is judged to be of a kind which is likely to attack lead-pipes, or when it actually flows through them impregnated with lead, a remedy may be found, either in leaving the pipes full of the water and at rest for three or four months, or by substituting for the water a weak solution of phosphate of soda, in the proportion of about a 25,000th part."

*e. Lake-water.*—This is a collection of rain, spring, and river-water, usually contaminated with putrefying organic matter.

*f. Marsh-water.*—This is similar to lake-water, except that it is altogether stagnant, and is more loaded with putrescent matter. The sulphates in sea and other waters are decomposed by putrefying vegetable matter, with the evolution of sulphuretted hydrogen; hence the intolerable stench from marshy and swampy grounds liable to occasional inundations from the sea.

The ordinary modes of purifying common water are *filtration, ebullition, distillation,* and by the addition of *chemical agents.* We shall now pass on to the other alimentary principles, and, passing over the *mucilaginous,* the *saccharine,* the *amylaceous,* the *ligneous,* and the *pectinaceous principles,* as presenting but little that is new, we come to the *acidulous alimentary principle.* This principle has been admitted by the author for two reasons: the *first* is, that vegetable acid constitutes one of the ingredients of our foods. Fruits and succulent herbs, in both of which vegetable acid exists, have always been employed as food. Acetic acid, obtained by the acetous fermentation of wine, was in very early use. "Vinegar, either by accident or design, (says Dr. Prout,) has been employed by mankind in all ages, in greater or less quantity, as an aliment; or it has been formed artificially from certain bodies, with the view to alimentary purposes."

The *second* reason is, that the employment of vegetable acid as an aliment, is necessary for the preservation of health. At least it becomes pretty clearly established that the "complete and prolonged abstinence from succulent vegetables or fruits, or their preserved juices, as articles of food," is a cause of scurvy; and various "circumstances render it probable that the antiscorbutic virtue of succulent vegetables or fruits depends on the organic acids, or on some salt that enters the system only in combination with such acids. The latter supposition is the more probable, because the acids, pure, have much less efficacy in preventing scurvy than the vegetable juices from which they are derived. Lemon-juice evaporated to the consistence of a syrup, as originally recommended by Dr. Lind, was found very inferior to the fresh fruit; and the crystallised acid, after being extensively tried, was renounced in favour of the juice preserved simply by the addition of a certain proportion of spirit." All vegetable acids, however, which can be taken as food, are not equally efficacious; for though it may be admitted that lemon-juice is a valuable antiscorbutic, the same cannot be said of vinegar; the united observations of Drs. Lind, Gilbert Blane, and Trotter, having shown that the liberal use of vinegar by sailors did not prevent the appearance, nor check the progress, of scurvy. The author now proceeds the organic acids in most frequent use: commencing with—

1st, *Acetic Acid,* or the *Acid of Vinegar.* Anhydrous, or real acetic acid, as it exists in some acetates, has the following composition, $C_4H_3O_3$. *Glacial or Crystallisable Acetic Acid,* the strongest procurable, contains one equivalent of water. Its formula is $C_4H_3O_3 + Aqua$.

*Pyroligneous Acid,* called also *Wood-vinegar,* or *White-vinegar,* is obtained by the distillation of wood. When pure, it consists of acetic acid and water only.

The *common vinegar* of the shops is procured by subjecting an infusion of malt, or of a mixture of milk and raw barley, to the acetous fermentation. Hence it is commonly termed *Malt-vinegar.*

*Wine-vinegar,* or *French-vinegar,* is obtained from wines of inferior quality. It is of two kinds, *white* and *red*. *White* wine vinegar is usually preferred, as keeping better. The constituents of wine vinegar are very similar to those of malt vinegar. It contains a little bitartrate and sulphate of potash. Wine vinegar may be distinguished from malt vinegar by ammonia, which occasions in the former a purplish precipitate, but not in the latter.

Distilled vinegar is usually superseded in the shops by dilute pyroligneous acid; but this imitation has not so fragrant an odour as the genuine article.

Vinegar is used at the table as a condiment, on account of its agreeable flavour and refreshing odour. Taken in small quantities, it is quite wholesome, allaying thirst and checking præternatural heat. The habitual use of it is injurious, and, by disturbing the function of the stomach, may give rise to leanness. It is in repute with young ladies for diminishing obesity. But the following case, from Portal, quoted by Giacomini, shews the ill-consequences of employing it for this purpose:—

"A few years ago a young lady, in easy circumstances, enjoyed good health; she was very plump, had a good appetite, and a complexion blooming with roses and lilies. She began to look on her plumpness with suspicion; for her mother was very fat, and she was afraid of becoming like her. Accordingly she consulted a woman, who advised her to drink a small glass of vinegar daily: the young lady followed her advice, and her plumpness diminished. She was delighted with the success of the remedy, and continued it for more than a month. She began to have a cough; but it was dry at its commencement, and was considered as a slight cold, which would go off. Meantime, from dry it became moist; a slow fever came on, and a difficulty of breathing; her body became lean, and wasted away; night sweats, swelling of the feet and of the legs, succeeded, and a diarrhœa terminated her life. On examination all the lobes of the lungs were found filled with tubercles, and somewhat resembling a bunch of grapes."

We shall now pass on to the *Proteinaceous Alimentary Principle*. Several organic substances, both animal and vegetable, which are employed as aliments, contain as their basis, or at least yield, the substance called by Mulder *Proteine*. They may therefore be regarded as modifications of one another, or of *proteine*. The author has accordingly grouped them under the head of the proteinaceous alimentary principle. This group corresponds very nearly with that called by Dr. Prout the *Albuminous Alimentary Principle*. It differs, however, in not comprehending gelatinous substances, which the author deemed it advisable to form into a distinct group. The following is the analysis of proteine, as given by its discoverer Mulder.

| | From Fibrine. | From Ovalbumen. | From Vegetable Albumen. |
|---|---|---|---|
| Carbon . . | 55·44 | 55·30 | 54·99 |
| Hydrogen . | 6·95 | 6·94 | 6·87 |
| Nitrogen . | 16·05 | 16·02 | 15·66 |
| Oxygen . . | 21·56 | 21·74 | 22·48 |
| Proteine . | 100·00 | 100·00 | 100·00 |

Liebig has deduced the following formula for the representation of the composition of proteine:—

| | Atoms. | Eq. Wt. | Per Cent. |
|---|---|---|---|
| Carbon . . | 48 | 288 | 55·38 |
| Hydrogen . | 36 | 36 | 6·92 |
| Nitrogen . | 6 | 84 | 16·16 |
| Oxygen . | 14 | 112 | 21·54 |
| Proteine . . | 1 | 520 | 100·00 |

Proteine does not exist, as such, in organised beings. Combined with small quantities of mineral or organised substances (sulphur, phosphorus, potash, soda, common salt, and phosphate of lime), it constitutes fibrine, albumen, and caseine, both animal and vegetable.

Fibrine, albumen, and caseine, contain, besides proteine, sulphur, and phosphorus, a quantity of *saline matter*, and hence, when burned, they leave ashes (composed principally of phosphate of lime and alkaline salts). The dietetical properties of pure proteine have not yet been ascertained. The proteinaceous compounds constitute the plastic elements of nutrition. According to Liebig, they are produced by vegetables only, and cannot be formed by animals, " although the animal organism possesses the power of converting one modification of proteine into another, fibrine into albumen, or *vice versâ*, or both into caseine, &c. In this point of view, the vegetable forms of proteine, vegetable albumen, fibrine, and caseine, become signally important, as the only sources of proteine for animal life, and consequently of nutrition, strictly so called—that is, the growth in mass of the animal body."*

Proteinaceous aliments are obtained from both animals and vegetables, and it will, therefore, be convenient to consider them under two distinct groups, though Liebig states, that animal and vegetable fibrine, animal and vegetable albumen, and animal and vegetable caseine, are respectively identical in every particular.

1. *Animal Proteinaceous Principles.*—This sub-group comprehends Fibrine, Albumen, and Caseine.

*a. Fibrine; Animal Fibrine.*—The fibrine is contained in solution in the circulating blood, but coagulates when this fluid is drawn from the body, forming with the colouring particles the *clot* or *crassamentum.* In the solid state it constitutes the basis of muscular fibre. It forms, therefore, the principal constituent of the fleshy or lean parts of animals. It is also found in some other animal tissues.

*Quantity of Fibrine in Animal Substances.*

| *100 Parts.* | *Fibrine.* | | *Authority.* |
|---|---|---|---|
| Blood of the Hog . . . . . | 0·46 | | Andral, Gavarret, and Delafond. |
| " " Ox . . . . . | 0·37 | | |
| " " Sheep . . . | 0· 3 | | |
| Beef (muscle of) . . . . | 20·0 | *including albumen.* | Brande. |
| Veal (ditto) . . . . . . | 19·0 | | Ditto. |
| Mutton (do.) . . . . . . | 22 | | Ditto. |
| Pork (do.) . . . . . . | 19 | | Ditto. |
| Chicken (do.) . . . . . . | 20 | | Ditto. |
| Cod (do.) . . . . . . | 14 | | Ditto. |

* Turner's Chemistry, 7th edition.

| 100 *Parts.* | *Fibrine.* | *Authority.* |
|---|---|---|
| Haddock (do.) . . . . . . | 13 | Brande. |
| Sole (do.) . . . . . . | 15 | |
| Calf's Sweetbread (Thymus) } . . . | 8 | Morin. |

Fibrine (as beef-steak, &c.) is readily soluble in an artificial digestive liquid.* It is also speedily dissolved in the living stomach, and is generally considered, even by dyspeptics, as being easy of digestion. It is an important element of nutrition, and yields fibrine, albumen, and caseine, as well as the tissues composed of those substances. Alone, however, it is incapable of supporting life, except for a very limited period. Magendie mentions, as a most singular circumstance, that animals who took regularly for two months from 500 grammes (1lb. 4oz. 37 grains troy,) to double that quantity of fibrine daily, died of inanition; and, on a post-mortem examination, it was found that the blood had almost entirely disappeared. "Notwithstanding," says Magendie, "the care we took to collect it (the blood) a few minutes after death, scarcely 15 grains troy of fibrine could be obtained."

*b. Albumen; Animal Albumen.*—This substance constitutes the most important part of animal foods. The albumen, both of the egg *(ovalbumen,)* and of the serum of the blood (*seralbumen,*) is liquid. But the albumen of flesh, glands, and viscera of animals, is solid.

Albumen is highly nutritious, and when either raw or lightly boiled, is easy of digestion; but when boiled hard, or especially when fried, its digestibility is considerably impaired. Albumen, says Liebig, must be considered as the true starting point of all the animal tissues. Still, animals cannot subsist solely on albumen. After a few days use of it they refuse to take it, preferring to suffer the most violent pangs of hunger rather than eat it, and ultimately they die of inanition.

*c. Animal Caseine; Caseum; Lactalbumen; Curd.*—This is the coagulable matter of milk, and is closely allied to albumen, of which it may be regarded as a modification.

The quantity of caseine contained in different kinds of milk varies considerably. Caseine, like albumen and fibrine, is a proteinaceous substance, differing from the two latter in containing no phosphorus. Coagulated caseine, deprived of whey by pressure, and usually mixed with more or less of butter, constitutes *cheese;* the richness of which is in proportion to the quantity of butter present. Rich cheese, when toasted, undergoes a kind of semifusion, and becomes soft and viscid. *Stilton cheese* is prepared from milk to which cream is added. *Cheshire* and the best *Gloucester cheeses* are made from unskimmed milk. *Suffolk* and *Parmesan cheeses* are prepared from skim-milk. Annotta is often employed, as a colouring agent, in the preparation of cheese. Salt is used to preserve it as well as to improve the flavour and add to the weight.

Cheese is subject to the attacks of both animals and vegetables. The Fly called *Musca* (*Tephritis*) *putris* deposits its leaping larvæ or maggots (called *hoppers* or *jumpers*) on cheese. The *cheese-mite (Acarus domesticus*

* Prepared by macerating the lining membrane of the 4th stomach of the calf in water, to which a few drops of hydrochloric acid are to be added.

is another animal of frequent occurrence. The *Mould* of cheese is composed of minute fungi. *Blue Mould* is the *Aspergillus glaucus* of Berkeley; while *Red Cheese-mould* is the *Sporendonema Casei* of the same authority.

Caseine is highly nutritious, constituting a plastic element of nutrition, by which, in the young mammal, the development of the tissues is effected.

"The young animal," says Liebig, "receives in the form of caseine the chief constituent of the mother's blood. To convert caseine into blood no foreign substance is required, and in the conversion of the mother's blood into caseine, no elements of the constituents of the blood have been separated. When chemically examined, caseine is found to contain a much larger proportion of the earth of bones than blood does, and that in a very soluble form, capable of reaching every part of the body. Thus, even in the earliest period of its life, the development of the organs, in which vitality resides, is, in the carnivorous animal, dependent on the supply of a substance, identical in organic composition with the chief constituents of its blood."

2. *Vegetable Proteinaceous Substances.*—According to Liebig, vegetables contain proximate principles, which are not only similar to, but absolutely identical with, the fibrine, albumen, and caseine, of animals; and he, therefore, denominates them respectively, vegetable fibrine, vegetable albumen, and vegetable caseine. There is also a fourth proteinaceous vegetable principle called glutine, or pure gluten.

*a. Vegetable Fibrine.*—This principle is most abundant in the seeds of the cereal grasses, as wheat, rye, barley, oats, maize and rice. It exists, also, in buckwheat, and in the juice of grapes. It is also found in the newly-expressed juices of most vegetables, as of carrots, turnips, &c. It differs from vegetable albumen, and vegetable caseine, in being insoluble in water.

*b. Vegetable Albumen.*—This, like vegetable fibrine, is a constituent of the seeds of the cereal grasses, as of wheat. In the preparation of raw gluten from wheaten dough, it is washed away along with the starch. It is found in great abundance in the oily seeds, as almonds &c.

Most vegetable juices contain a considerable quantity of it. This principle differs from the vegetable fibrine in being soluble in water, and from vegetable caseine in coagulating when heated.

*c. Vegetable Caseine.*—This is chiefly found in leguminous seeds, as beans, peas, lentils; and has, in consequence, been termed *Legumine.* The oily seeds, such as almonds, also contain it along with albumen. It differs from vegetable fibrine in being soluble in water; and from vegetable albumen in not coagulating when its aqueous solution is heated.

*d. Pure Gluten.*—By washing wheaten dough with a stream of water, the gum, sugar, starch, and vegetable albumen are removed; while a ductile, tenacious, elastic, gray mass is left, usually called *gluten.*

Gluten is easy of digestion, is highly nutritious, and alone it is capable of the prolonged nutrition of animals.

*The Gelatinous Alimentary Principle.*

Dr. Prout comprehends gelatine among albuminous aliments. He considers it to be a modification of albumen, or as the least perfect kind of albuminous matter existing in animal bodies.

But, according to our author, gelatine and albumen, and the proteinaceous and albuminous tissues respectively differ in their chemical properties and composition. Though it is probable that in the animal system gelatinous tissues are formed out of proteine compounds, chemists have hitherto totally failed to convert albumen into gelatine, or *vice versâ.* Again, as the composition of proteine compounds is identical with the flesh and blood of animals, while that of the gelatinous tissues is not, it follows that the nutritive qualities of the proteinaceous and gelatinous tissues cannot be identical. Hence the propriety of separating gelatinous from albuminous aliments.

Albuminous or proteinaceous tissues are insoluble in water, and by boiling become hard. Gelatinous tissues, on the other hand, yield, by boiling, a substance called *gelatine,* which is soluble, and forms with water a tremulous mass, termed *jelly* (*animal jelly).*

The digestibility of the different varieties and forms of gelatinous matter is not uniform. *Calf's-foot jelly,* when fresh prepared, is found to be readily digested even by invalids and dyspeptics. *Isinglass jelly,* when fresh prepared from isinglass of good quality, and also *Hartshorn jelly,* are probably equally easy of digestion. Other forms, however, of gelatinous matter, are more difficult of digestion. Thus *very hard gelatinous tissues,* as *tendons,* require a larger quantity of gastric juice, and a longer time for their complete digestion. *Gelatinous liquids,* when very weak, or which are obtained by means of a high temperature, or which are obtained from tissues containing fat or other matters apt to become rancid, readily disturb the functions of the stomach or intestines. *Soups, hashes,* and *stews,* all of which contain gelatine, are obnoxious to the digestive organs of dyspeptics and invalids, principally from the presence of fatty and other substances difficult of digestion.

The times required for the digestion of various substances, as ascertained by Dr. Beaumont, are as follows ;—

| Articles of Diet. | Mean Time of Chymification | | | | | |
|---|---|---|---|---|---|---|
| | In Stomach. | | | In Phials. | | |
| | Preparation. | H. | M. | Preparation. | H. | M. |
| Calf's-foot jelly .. | Boiled .. | 1 | 0 | | | |
| Isinglass jelly .. .. | Boiled .. | 1 | 0 | | | |
| Gelatine .. .. .. | Boiled .. | 2 | 30 | Boiled .. .. | 4 | 45 |
| Aponeurosis .. .. | Boiled .. | 3 | 0 | Boiled .. .. | 6 | 30 |
| Cartilage .. .. .. | Boiled .. | 4 | 15 | —— | | — |
| Cartilage .. .. .. | — | — | | Divided .. | 12 | 0 |
| Tendon .. .. .. | Boiled .. | 5 | 30 | Masticated .. | 12 | 45 |
| Tendon of young beef | — | — | | Entire piece.. | 24 | 0 |
| Bones, beef, solid .. | — | — | | Entire piece.. | 80 | 0 |
| " hogs, solid .. | — | — | | Entire piece.. | 80 | 0 |

A gelatinous substance, though possessing some degree of nutritive power, cannot alone sustain animal life; but when taken in conjunction with other alimentary substances, takes part in the nutrition of the body. Different gelatinous substances, however, are unequally nutritive. Thus gelatine is less nutritive than the bone which yields it. It has been found that dogs fed solely on raw bones and water for three months, continued in perfect health, and lost none of their weight by the use of this kind of food. Now, as by boiling in water, the cartilaginous tissue of bone is resolved into gelatine principally, it follows that a gelatinous tissue, (that is, a tissue which by boiling is resolved into gelatine,) contributes to the nutrition of the body; though it cannot be said to be the exclusive agent in this process, since bones contain other alimentary principles (such as fatty and albuminous matters) besides the earthy salts and the substance which is resolvable into gelatine.

An exclusive diet of beef tendon and water is incapable of effecting perfect nutrition,—this has been proved by experiment.

Gelatine extracted from bones was refused by dogs,—by some from the first, by others after once or twice using it. They preferred enduring the pangs of hunger to eating it. Seasoned gelatine, prepared for the use of man, was eaten for a few days, and then refused; the animals dying of starvation on the twentieth day. Hence it follows, that animals cannot be nourished on gelatine exclusively. We are not justified, however, in saying, that gelatine, conjoined with other elementary substances, does not assist in nutrition. Liebig has suggested, that the nourishing powers of gelatine are confined to the gelatinous tissues; for as proteine cannot be obtained from gelatine, the latter can serve neither for the formation of blood, nor for the reproduction and growth of albuminous and fibrinous tissues. It is therefore probable, he thinks, that gelatine when taken in the dissolved state, is again converted in the body into cellular tissue, membrane and cartilage. And when the powers of nutrition in the whole body are affected by a change of the health, then, even should the power of forming blood remain the same, the organic force by which the constituents of the blood are transformed into cellular tissue and membranes, must necessarily be enfeebled by sickness. In the sick man, the intensity of the vital force, its powers to produce metamorphoses, must be diminished, as well in the stomach as in all other parts of the body. In this condition, the uniform experience of practical physicians shows that gelatinous matters, in a dissolved state, exercise a most decided influence on the state of the health. Given in a form adapted for assimilation, they serve to husband the vital force, just as may be done, in the case of the stomach, by due preparation of the food in general. Such are the ingenious views of Liebig, with which our author, by the way, does not seem fully to coincide.

We find our analysis of this interesting work, has transgressed the bounds originally designed by us. With respect to its merits, we find ourselves called on to state, that we consider it a careful compilation of the opinions of the best writers on the various subjects of which it treats. The ingenious and philosophical views of Liebig have been brought into ample requisition. We fear, however, that the book will never become a popular work. The symbolical language in which the most interesting parts of it are presented, is totally unintelligible, not only to the general reader, but we fear, also, to many, very many, of our professional brethren.

